# Pointwise monotonicity of heat kernels

**DOI:** 10.1007/s13163-021-00417-8

**Published:** 2021-12-13

**Authors:** Diego Alonso-Orán, Fernando Chamizo, Ángel D. Martínez, Albert Mas

**Affiliations:** 1 Institute für Angewandte Mathematik, Universitat Bonn, Endenicher Allee 60, Bonn, 53115 Germany; 2 Instituto de Ciencias Matemáticas (CSIC-UAM-UC3M-UCM), Departamento de Matemáticas, (Universidad Autónoma de Madrid), Madrid, 28049 Spain; 3 Departament de Matemàtiques, Universitat Politècnica de Catalunya, Campus Diagonal Besòs, Edifici A (EEBE), Av. Eduard Maristany 16, Barcelona, 08019 Spain

**Keywords:** Heat kernel, Maximum principle, Fractional Laplacian, Pointwise inequalities, 35K08, 35B50

## Abstract

In this paper we present an elementary proof of a pointwise radial monotonicity property of heat kernels that is shared by the Euclidean spaces, spheres and hyperbolic spaces. The main result was discovered by Cheeger and Yau in 1981 and rediscovered in special cases during the last few years. It deals with the monotonicity of the heat kernel from special points on revolution hypersurfaces. Our proof hinges on a non straightforward but elementary application of the parabolic maximum principle. As a consequence of the monotonicity property, we derive new inequalities involving classical special functions.

## Introduction

The heat equation is one of the quintessential mathematical models for physical phenomena. Over the years, several properties of this equation had been studied from different points of view, including for instance: probabilistic, geometric and physical. In this paper we deal with the fundamental solution of the heat equation, namely, the heat kernel. In particular, we will focus on certain generalizations, due to Cheeger and Yau, of the following intuitive result

### Theorem 1

([1–4]) Let *M* be Euclidean space ${\mathbb {R}}^n$, the sphere ${\mathbb {S}}^n$ or the hyperbolic space ${\mathbb {H}}^n$. Then, for any fixed $x\in M$ and time $t\in (0,\infty )$, the heat kernel *G*(*x*, *y*, *t*) is a strictly decreasing function of the geodesic distance *d*(*x*, *y*).

First, note that by means of Fourier analysis one can provide an explicit expression of the heat kernel in the Euclidean space ${{\mathbb {R}}}^n$, namely

$$\begin{aligned} G(x,y,t)=\frac{1}{(4\pi t)^{n/2}}\exp \left( -\frac{|x-y|^2}{4t}\right) , \end{aligned}$$

where the assertion of Theorem 1 follows trivially. However, explicit expressions like the above are rare. The hyperbolic spaces are exceptions, e.g. for the hyperbolic plane one gets the following representation

$$\begin{aligned} G(x,y,t)=\frac{\sqrt{2}e^{-t/4}}{(4\pi t)^{3/2}}\int _{d(x,y)}^{\infty }\frac{\beta e^{-\beta ^2/4t}}{\sqrt{\cosh \beta -\cosh (d(x,y))}}\,d\beta . \end{aligned}$$

In general, distinguishing odd and even dimensional cases, rather involved formulas are available (cf. [2]). The case of the sphere does not share this good fortune, but one can use an identity^1^ expressing the derivative of the kernel in terms of the heat kernel in smaller dimensions to prove the monotonicity by an induction argument (the base case can be found in [2, Section 6.3]). Recently, Nowak, Sjögren and Szarek [4] provided sharp estimates for the heat kernel on the sphere ${\mathbb {S}}^n$ that imply Theorem 1 in that specific case.

Here we propose a neat, direct and elementary proof of Theorem 1 which is built on a delicate application of the parabolic maximum principle. Interestingly enough, the same arguments also apply to more general situations described below, of which Theorem 1 is a beautiful particular case.

### Theorem 2

Let $M\subseteq {\mathbb {R}}^n$ be a smooth, compact and connected hypersurface of revolution around the $x_n$ axis without boundary. If *x* is a point of intersection of *M* and the $x_n$ axis, then the associated heat kernel *G*(*x*, *y*, *t*) strictly decreases as a function of the geodesic distance *d*(*x*, *y*) for any fixed $t>0$.

### Remark 1

The same proof covers the noncompact situation even in an intrinsic geometric setting beyond hypersurfaces of ${{\mathbb {R}}}^n$. In connection with this, recall that a celebrated theorem by Hilbert states that a complete regular surface of constant negative curvature, like ${\mathbb {H}}^2$, cannot be isometrically immersed in ${{\mathbb {R}}}^3$.

### Remark 2

When we compare our approach to that of Cheeger and Yau [3], we strongly believe that ours is more PDE friendly and falls within a certain canon. Both proofs rely on the maximum principle, nevertheless. The proof in [3] is more technically demanding, namely it employs 1-forms and the small time asymptotics of the heat kernel. Furthermore, it relies on a certain contradiction (obtained using an integral quantity that is proved to be zero) while ours is more direct.

Theorem 2 can be proven even in more general settings. Let us introduce some definitions before stating this. In a complete Riemannian manifold *M* of dimension *n*, a point *p* is called a pole if its cut locus is empty (see [5] for the definition of the cut locus). A manifold is said to be spherically symmetric (around *p*) if in terms of the geodesic polar coordinates $(\rho ,\sigma )\in (0,\infty )\times {\mathbb {S}}^{n-1}$, its Riemannian metric $ds^{2}$ of *M* has the form $ds^{2}=d\rho ^2+A^2(\rho )d\sigma ^2$. In other words, the rotations around the origin in $T_p(M)$ become isometries of *M* under the exponential map.^2^ Finally, we need some control on the volume of the surface of balls in order to apply the maximum principle.

### Theorem 3

Let *M* be a complete spherically symmetric manifold with bounded curvature. Let *S* be the volume of the boundary of the ball of radius $\rho$ in *M* centered on a pole *p* and suppose that $\frac{\partial ^{2}}{\partial \rho ^{2}}\log (S(\rho ))$ is bounded from above. Then the heat kernel *G*(*p*, *y*, *t*) based on the pole *p*, satisfies $\int _M G(p,y,t)\; dy=1$ and it is a strictly decreasing function of the geodesic distance *d*(*p*, *y*) for any fixed $t>0$.

### Remark 3

We have recently been aware of an extension of the results in [4] by the same authors where they provide sharp bounds for the heat kernels on all compact rank-one symmetric spaces, cf. [7].

Our last result, also known to Cheeger and Yau, tackles the Dirichlet and Neumann heat kernels, $G_D$ and $G_N$ respectively, of a smooth hypersurface of revolution $M\subseteq {{\mathbb {R}}}^n$ with boundary. Recall that $G_D$ is the fundamental solution for the Dirichlet heat operator $\partial _t-\Delta _M$ (where we denoted by $-\Delta _M$ the positive Laplace–Beltrami operator on *M*) with the Dirichlet boundary condition, that is, for a fixed $x\in M$, $G_D(x,y,t)$ is the function in (*y*, *t*) satisfying

$$\begin{aligned} {\left\{ \begin{array}{ll} (\partial _t-\Delta _M)G_D(x,y,t)=0, &{}(y,t)\in M\times (0,+\infty ),\\ G_D(x,y,t)=0, &{}(y,t)\in \partial M\times (0,+\infty ),\\ G_D(x,y,0)=\delta _x(y), &{} y\in M, \end{array}\right. } \end{aligned}$$

where $\delta _x$ is the Dirac delta based on *x*. Similarly, for $G_N$ with the Neumann boundary condition, that is, for a fixed $x\in M$, $G_N(x,y,t)$ is the function in (*y*, *t*) satisfying

$$\begin{aligned} {\left\{ \begin{array}{ll} (\partial _t-\Delta _M)G_N(x,y,t)=0, &{}(y,t)\in M\times (0,+\infty ),\\ \frac{\partial }{\partial n}\,G_N(x,y,t)=0, &{}(y,t)\in \partial M\times (0,+\infty ),\\ G_N(x,y,0)=\delta _x(y), &{} y\in M. \end{array}\right. } \end{aligned}$$

### Theorem 4

Let $M\subseteq {\mathbb {R}}^n$ be smooth and connected hypersurface of revolution around the $x_n$ axis with boundary $\partial M\ne \emptyset$. If *x* is a point of intersection of the relative interior of *M* and the $x_n$ axis, then the associated heat kernel with Dirichlet boundary condition (resp. Neumann boundary condition) $G_D(x,y,t)\ ($resp. $G_N(x,y,t))$ strictly decreases as a function of the geodesic distance *d*(*x*, *y*) for any fixed time $t>0$.

### Remark 4

An analogous result in the case of the Dirichlet heat kernel on a geodesic ball inside a *n*-dimensional simply connected space form of constant sectional curvature was already known (cf. [2, Section 8.3]). Theorem 4 has also been settled (cf. [3, Section 2]) for model manifolds. The interest of these results originates from the possibility of comparing the fundamental solutions to the heat equation on general manifolds with heat kernels of model manifolds.

### Plan of the paper

The paper is organized along the following lines: in the next section we present the proofs of the obtained results where we demonstrate Theorem 2, sketch the proof of Theorem 3 and infer as a consequence Theorem 1. Section 3 is devoted to show some consequences regarding inequalities for special functions and some observations of pointwise properties of the fractional Laplace–Beltrami operator. The article ends raising a natural question that bonds the decreasing properties of the heat kernel from a point with its cut locus.

## Proof of the main results

Note that Theorem 1 is a direct corollary of Theorem 3. The main ideas in the proof of the latter are in that of Theorem 2. The whole argument relies on an application of the parabolic maximum principle.

### Proof of Theorem 2

Thanks to the symmetry around the $x_n$ axis, the heat kernel *G*(*x*, *y*, *t*) is a function $\Gamma$ only depending on the geodesic distance from *x* to *y*, $\rho =d(x,y)$, and the time variable *t*, that is, $G(x,y,t)=: \Gamma (\rho ,t)$. This symmetry also justifies expressing the homogeneous heat equation on *M* for smooth^3^ functions $u:(0,L)\times (0,+\infty )\rightarrow {{\mathbb {R}}}$ which depend only on the geodesic distance and the time variable as [6, Section 3.2]

1 $$\begin{aligned} \partial _t u(\rho ,t)-\partial _{\rho \rho }u(\rho ,t)-\partial _\rho \big (\log (S(\rho ))\big )\,\partial _\rho u(\rho ,t)=0 \end{aligned}$$

for all $\rho \in (0,L),\,t>0$, where *L* denotes the geodesic distance from *x* to its antipodal point, and $S(\rho )$ denotes the volume of the boundary of the ball of radius $\rho$ in *M* centered at *x*. Differentiating (1) with respect to $\rho$ we obtain

2 $$\begin{aligned} \partial _t (\partial _\rho u)-\partial _{\rho \rho }(\partial _\rho u)-\partial _{\rho \rho }\big (\log (S(\rho ))\big )\,(\partial _\rho u) -\partial _\rho \big (\log (S(\rho ))\big )\,\partial _\rho (\partial _\rho u)=0. \end{aligned}$$

Near *x* we see that $\partial _{\rho \rho }(\log (S(\rho )))$ behaves like in the euclidean case. This provides the approximation $-(n-2)\rho ^{-2}$ for $\rho$ near zero. By continuity and compactness we conclude that $\partial _{\rho \rho }(\log (S(\rho )))$ is bounded from above in (0, *L*) if *M* is compact. With this at hand, we define

3 $$\begin{aligned} \begin{aligned} \lambda&:=\textstyle {\sup _{\rho \in (0,L)}}\partial _{\rho \rho }\big (\log (S(\rho ))\big ),\\ c(\rho )&:=\lambda -\partial _{\rho \rho }\big (\log (S(\rho ))\big ),\\ v(\rho ,t)&:=e^{-\lambda t}(\partial _\rho u)(\rho ,t).\\ \end{aligned} \end{aligned}$$

By the previous considerations, we have that $\lambda \in {{\mathbb {R}}}$ and $c\ge 0$. Now, since $\partial _t (\partial _\rho u)= e^{\lambda t}(\lambda v+\partial _t v)$, from (2) we get

4 $$\begin{aligned} \begin{aligned} 0&=\lambda v+\partial _t v-\partial _{\rho \rho }v-\partial _{\rho \rho }\big (\log (S(\rho ))\big )\,v -\partial _\rho \big (\log (S(\rho ))\big )\,\partial _\rho v\\&=\partial _t v-\partial _{\rho \rho }v +b\partial _\rho v+cv=(\partial _t+{\mathcal {L}})v, \end{aligned} \end{aligned}$$

where we have set $b(\rho ):=-\partial _\rho (\log (S(\rho )))$ and ${\mathcal {L}}:=-\partial _{\rho \rho }+b\partial _{\rho }+c$.

Our first target is to show that $\Gamma (\cdot ,t)$ is a nonincreasing function on [0, *L*] for all $t>0$. In order to do so, let us regularize the heat kernel *G* employing a family of functions $\{\chi _{\epsilon }(x,\cdot )\}_{\epsilon >0}$ that satisfy the following properties: they are smooth, radially symmetric (i.e. $\chi _{\epsilon }(x,y)=: g_{\epsilon }(\rho )$), and decreasing from *x*, their integral over *M* equals one, and they concentrate around the fixed point $x\in M$ as $\epsilon$ tends to zero. Notice that this family is, in particular, an approximation of the Dirac delta centered at *x*. Let us now introduce the aforementioned regularization of the heat kernel

$$\begin{aligned} G_{\epsilon }(x,y,t):=\int _{M}\chi _{\epsilon }(x,z)G(z,y,t)\,d\sigma (z), \end{aligned}$$

where $d\sigma$ denotes the volume form on *M*. By construction, $G_{\epsilon }$ satisfies the heat equation in the variables (*y*, *t*) and the initial condition $\chi _{\epsilon }(x,\cdot )$. Moreover, since *G* is smooth for $t>0$ and $\chi _{\epsilon }$ is also smooth, we deduce that $G_{\epsilon }$ is smooth up to $t=0$. In addition, due to the radial symmetry of *M* with respect to *x*, and of $\chi _\epsilon$, $G_{\epsilon }(x,y,t)$ only depends on the radial coordinate $\rho =d(x,y)$ emanating from *x* and on *t*, hence we can write $G_{\epsilon }(x,y,t)=:u_\epsilon (\rho ,t)$. In particular $2u_\epsilon (\rho ,t)=G_\epsilon (x,\exp _x(\rho \mathbf {e}_1),t)+G_\epsilon (x,\exp _x(-\rho \mathbf {e}_1),t)$. Then $\partial _\rho u_\epsilon (0,t)=0$ for $t>0$ and a similar argument at the antipodal point shows $\partial _\rho u_\epsilon (L,t)=0$. Therefore, if we set $v_\epsilon :=e^{-\lambda t}\partial _\rho u_\epsilon$, taking $u=u_\epsilon$ in (3) and $v=v_\epsilon$ in (4) we deduce that, for every $T>0$,

$$\begin{aligned} \left\{ \begin{array}{ll} \partial _t v_{\epsilon }+{\mathcal {L}}v_{\epsilon }=0 &{} \text {in }(0,L)\times (0,T),\\ v_{\epsilon }(0,t)= v_{\epsilon }(L,t)=0&{} \text {for\ all}\ t\in (0,T),\\ v_{\epsilon }(\cdot ,0)=\partial _\rho g_\epsilon \le 0 &{} \text {in}\ [0,\,{ L}]. \end{array}\right. \end{aligned}$$

Recalling that ${\mathcal {L}}=-\partial _{\rho \rho }+b\partial _{\rho }+c$ with $c\ge 0$, by the weak maximum principle [8, Theorem 9 in page 392] we see that $v_\epsilon \le 0$ in $[0,L]\times [0,T]$. Taking now $T\rightarrow +\infty$ we get that $\partial _\rho u_\epsilon (\cdot ,t) =e^{\lambda t}v_\epsilon \le 0$ for all $t>0$, which means that $u_\epsilon (\cdot ,t)$ is nonincreasing for all $t>0$ and all $\epsilon >0$. Finally, since $G_{\epsilon }(x,y,t)\rightarrow G(x,y,t)$ for $t>0$ as $\epsilon \rightarrow 0$, we have $\Gamma (\rho ,t)=\lim _{\epsilon \rightarrow 0}u_\epsilon (\rho ,t)$ and, thus, $\Gamma (\cdot ,t)$ is a nonincreasing function on [0, *L*] for all $t>0$.

Our goal now is to show that $\Gamma (\cdot ,t)$ is indeed a strictly decreasing function on [0, *L*] for all $t>0$. This will mean that, for every $t>0$, the heat kernel *G*(*x*, *y*, *t*) is strictly decreasing as a function of the geodesic distance $\rho =d(x,y)$ emanating from *x*, which is the claim of the theorem.

Take $u=\Gamma$ and $v=e^{-\lambda t}\partial _\rho u$ as in (3). Since *u* satisfies (1), we have $(\partial _t+{\mathcal {L}})v=0$ by (4). Also, we already know that $\partial _\rho \Gamma (\cdot ,t)\le 0$ for all $t>0$, which yields $v\le 0$ in $[0,L]\times (0,+\infty )$. Therefore, given $t_0>0$, if we set $w(\rho ,t):=v(\rho ,t+t_0)$, *w* solves

$$\begin{aligned} \left\{ \begin{array}{ll} \partial _t w+{\mathcal {L}}w=0 &{} \text {in }(0,L)\times (0,+\infty ),\\ w\le 0&{} \text {in }[0,L]\times [0,+\infty ). \end{array}\right. \end{aligned}$$

We will show that if $t_0$ is small enough then $w<0$ in $(0,L)\times (0,+\infty )$. Arguing by contradiction, assume that $w(r,\tau )=0$ for some $(r,\tau )\in (0,L)\times (0,+\infty )$. Since $w\le 0$ in $[0,L]\times [0,+\infty )$, we deduce that *w* attains a nonnegative maximum over $[0,L]\times [0,\tau ]$ at the interior point $(r,\tau )$. Then, since $c\ge 0$, the strong maximum principle [8, Theorem 12 in page 399] yields that $w=0$ in $[0,L]\times [0,\tau ]$, which leads to $\partial _\rho \Gamma =0$ in $[0,L]\times [t_0,t_0+\tau ]$. In particular, $\Gamma (\cdot ,t_0)$ is constant on [0, *L*]. By taking $t_0$ small enough in the definition of *w*, we get a contradiction with the fact that $\Gamma (\rho ,t)=G(x,y,t)$ tends to the Dirac delta centered at $x\in M$ as $t\rightarrow 0$ (and hence $\Gamma (\cdot ,t_0)$ is not constant for $t_0>0$ small enough).

Once we know that $w<0$ in $(0,L)\times (0,+\infty )$, we deduce that $\partial _\rho \Gamma <0$ in $(0,L)\times (t_0,+\infty )$ and, as $t_0$ can be chosen arbitrarily small, we conclude that $\partial _\rho \Gamma (\cdot ,t)<0$ for all $t>0$. Therefore, $\Gamma (\cdot ,t)$ is a strictly decreasing function on [0, *L*] for all $t>0$. The theorem is finally proven. $\square$

### Remark 5

The previous proof still applies dropping the compactness condition if we can assure that $\frac{\partial ^2}{\partial \rho ^2}\log (S(\rho ))$ remains bounded from above and decays at infinity. The proof of the next result requires to justify the decay condition for the noncompact case.

Next let us show the proof of Theorem 3:

### Proof of Theorem 3

The proof follows the same lines as the previous one. Since the boundedness of $\frac{\partial ^2}{\partial \rho ^2}\log (S(\rho ))$ is part of the hypothesis, we just need to provide an exponential decay of the radial derivative of $G(x,\cdot ,t)$. Under the geometric hypothesis in the statement Cheng, Li, and Yau have shown already that, for $t\in [0,T)$,

$$\begin{aligned} |\nabla G(x,y,t)|\le C_T t^{(n+1)/2}e^{-cd(x,y)^2/t}, \end{aligned}$$

for some constants $c, C_T>0$ independent on the distance *d*(*x*, *y*) (cf. Theorem 6, [9, Section 4] p. 1055). This is enough to ensure the decay at infinity to finish the argument. Observe that one may use the maximum principle on a space-time box $B(r)\times [0,T]$, then the maxima should be achieved at the boundary $t=0$. Indeed, the heat equation prevents a maximum to be achieved in $t=T$; on the other hand one can extend the box to be a band by letting *r* tend to infinity (i.e. $M\times [0,T]$) since the boundary terms tend to zero (due to the exponential decay at infinity model that the aforementioned bound provides). $\square$

### Remark 6

The proof of Theorem 4 is completely analogous to the one of Theorem 2. Indeed, for Dirichlet heat kernels one observes that $G_D(x,y,t)\ge 0$ in the interior and it vanishes at the boundary. This shows that its radial derivative is less than or equal to zero at the boundary, the rest of the proof is analogous. On the other hand, for the Neumann heat kernel the boundary replaces the antipodal point and the Neumann condition on the boundary replaces the radial derivative that vanishes at the antipodal point.

## Consequences regarding special functions and the fractional Laplace–Beltrami operator

In this section, we will present certain consequences of the aforementioned theorems regarding inequalities about classical special functions. To conclude we will also show some observations about pointwise properties of the fractional Laplace–Beltrami operator.

For that purpose, let us first recall that a function $F:{{\mathbb {R}}}^+\longrightarrow {{\mathbb {R}}}$ is said to be *completely monotonic* if $(-1)^nF^{(n)}>0$ for $n\in {{\mathbb {Z}}}_{\ge 0}$. The central result about them is Bernstein’s theorem [10, Section IV.12] stating that any completely monotonic function *F* can be written as $F(u)=\int _0^\infty e^{-tu}\; d\mu (t)$ for some nonnegative measure $d\mu$.

If *M* is as in Theorem 2, with the notation employed there, we have the spectral expansion (e.g. [11, Lemma 2.1])

$$\begin{aligned} G(x,y,t)= \sum _{k=1}^\infty e^{-\lambda _k t} \varphi _k(x)\varphi _k(y) \end{aligned}$$

with $\varphi _k$ normalized eigenfunctions of the Laplace–Beltrami operator $-\Delta _M$ corresponding to the eigenvalues $\lambda _k$. Combining the conclusion of Theorem 2 and Bernstein’s theorem we deduce

### Corollary 5

Let *M* and *x* be as in Theorem 2, and *F* a completely monotonic function such that the following series converges absolutely

$$\begin{aligned} \sum _{k=1}^\infty F(\lambda _k) \varphi _k(x)\varphi _k(y). \end{aligned}$$

Then it defines a strictly decreasing function of the geodesic distance *d*(*x*, *y*).

By definition, the kernel of the heat equation with the spectral fractional Laplace–Beltrami operator is (see [12] for the ${\mathbb {S}}^2$ case)

$$\begin{aligned} G(x,y,t)= \sum _{k=1}^\infty e^{-\lambda _k^\alpha t} \varphi _k(x)\varphi _k(y) \quad \text {with} \ 0<\alpha \le 1. \end{aligned}$$

As the function $F(x)= \exp (-x^\alpha )$ is completely monotonic for $0<\alpha \le 1$, we conclude at once

### Corollary 6

For *M* and *x* as in Theorem 2, consider the fractional heat equation $u_t+(-\Delta _{M})^\alpha u=0$ with $0<\alpha \le 1$ and $(-\Delta _{M})^\alpha$ the spectral fractional Laplace–Beltrami operator on *M*. Then, its fundamental solution decreases as a function of the geodesic distance *d*(*x*, *y*).

This generalizes to other operators if the symbol has a completely monotonic derivative because a simple calculation [13] shows that if $F'(x)$ is completely monotonic then $\exp (-tF(x))$ is also completely monotonic for all $t>0$.

For some particular manifolds *M*, there exists a full description of the eigenvalues and eigenfunctions in terms of special functions. In these cases, taking derivatives, Corollary 5 can be read as the positivity of some weighted sums of special functions. We exploit this relation for a compact manifold, for a manifold with boundary and for a noncompact manifold to get some inequalities involving averages of some classic special functions which seem to be new.

We start with the Legendre polynomials $P_n$. Many authors have considered inequalities involving $P_n$ and its derivatives (see [14]). Arguably the cleanest is Fejér’s inequality $\sum _{n=0}^N P_n(x)> 0$ for $-1<x<1$ that we still find in recent research [15]. There are several classic results by Hilb, Stieltjes, and other authors [14] showing intricate oscillations of $P_n(x)$ and $P_n'(x)$ when *n* and *x* vary and it is a theoretical obstacle to get general simple inequalities. Here we use Corollary 5 and the link between the Legendre polynomials and spectral expansions on the sphere to get a neat result.

### Corollary 7

Let *F* be a completely monotonic function such that $F(x)=O\big (x^{-\sigma }\big )$ for some $\sigma >2$. Then,

$$\begin{aligned} \sum _{n=0}^\infty (2n+1)P_n'(x)F\big (n(n+1)\big ) \ge 0 \quad \text {for}\ -1\le x\le 1. \end{aligned}$$

Thanks to the identity $(1-x^2)(2n+1)P_n'(x)=n(n+1)\big (P_{n-1}(x)-P_{n+1}(x)\big )$, see [16, 8.914.2], the previous corollary can also be rephrased in terms of a sum of $P_n$.

### Proof

It is well known [17, Section VII.5] that the eigenvalues of the Laplace–Beltrami operator on ${{\mathbb {S}}}^2$ are $\lambda _n=n(n+1)$ with $n\in {{\mathbb {Z}}}_{\ge 0}$ and $\lambda _n$ has multiplicity $2n+1$ with corresponding eigenfunctions $P_n^m(\cos \theta )\cos (m\varphi )$ with $0\le m\le n$ and $P_n^m(\cos \theta )\sin (m\varphi )$ with $0< m\le n$, where $P_n^m$ are the associate Legendre polynomials and $(\theta ,\varphi )$ are the usual spherical coordinates. Using the spherical harmonic addition theorem [16, 8.814] we have that the series in Corollary 5 is

$$\begin{aligned} \sum _{n=0}^\infty (2n+1) P_n(\cos \theta ) F\big (n(n+1)\big ) \end{aligned}$$

(see [17, Section VII.5.4] for the details) where $0\le \theta \le \pi$ gives the geodesic distance. From the bounds $|P_n(\cos \theta )|\le 1$ and $|P_n'(\cos \theta )|\le P_n'(1)=n(n+1)/2$ (see [14, (7.33.8)]) and our hypothesis $F(x)=O\big (x^{-\sigma }\big )$, the series can be differentiated term by term. The result follows because Corollary 5 implies that it is decreasing in $\theta$. The inequality at the end points follows by continuity. $\square$

Now we are going to deduce an inequality for a sum of Bessel functions. It is not strictly a consequence of Corollary 5 because we are going to impose Dirichlet conditions but the proof keep the same scheme appealing to Theorem 4 instead of Theorem 2.

### Corollary 8

Let *F* be a completely monotonic function such that $F(x)=O\big (x^{-\sigma }\big )$ for some $\sigma >5/4$. Then,

$$\begin{aligned} \sum _{n=1}^\infty \frac{z_n J_1(z_n r)}{J_1^2(z_n)} F\big (z_n^2\big ) \ge 0 \quad \text {for}\ 0\le r\le 1, \end{aligned}$$

where $J_1$ is the Bessel function of order 1 and $\{z_n\}_{n=1}^\infty$ is the sequence of positive zeros of $J_0$, the Bessel function of order 0.

### Proof

Consider the heat equation $u_t-\Delta u=0$ in the unit disk $D\subset {{\mathbb {R}}}^2$ under the Dirichlet boundary condition $u(\partial D,t)=0$. It is well known that the radial eigenfunctions of $\Delta u$ satisfying the boundary condition are $J_0(z_nr)$. Then by separation of variables the radial solutions are of the form [18, Section II.34]

$$\begin{aligned} u(r,t)= \sum _{n=1}^\infty a_n e^{-z_n^2t}J_0(z_n r). \end{aligned}$$

On the other hand, we have the Fourier–Bessel series expansion [18, Section I.17]

$$\begin{aligned} f(x)= \sum _{n=1}^\infty a_n J_0(z_n x) \quad \text {with}\ a_n=\frac{2}{J_1^2(z_n)} \int _0^1sf(s)J_0(z_n s)\; ds. \end{aligned}$$

To compute the fundamental solution we can choose the following approximation of the identity: $f(r)=(\pi \epsilon ^2)^{-1}$ if $0\le r<\epsilon$ and 0 otherwise. It is clear that formally we get the coefficients $a_n = \pi ^{-1} J_1^{-2}(z_n)$ when $\epsilon \rightarrow 0^+$, simply using $J_0(0)=1$. Then the fundamental solution should be

$$\begin{aligned} u(r,t)= \sum _{n=1}^\infty \frac{J_0(z_n r)}{\pi J_1^2(z_n)} e^{-z_n^2t}. \end{aligned}$$

By Theorem 4, for any given *t*, it defines a strictly decreasing function of $r\in (0,1)$. Using Bernstein’s theorem, we can replace $e^{-z_n^2t}$ by $F(z_n^2)$ and the result, under our hypothesis on *F*, is differentiable term by term because $\sqrt{t}J_0(t)$, $n/z_n$ and $\big (\sqrt{z_n}J_1(z_n)\big )^{-1}$ are uniformly bounded, indeed the last two sequences tend to $1/\pi$ and to $-\sqrt{\pi /2}$, respectively [16, 8.548, 8.541.1]. This can be used to show, by direct computation, that *u*(*r*, *t*) is indeed the fundamental solution we were seeking. The derivative of the resulting expression must be nonpositive and using $J_0'=-J_1$, the proof is complete. $\square$

We finish with a result involving the classical Mehler (conical Legendre) function $P_{-1/2+iv}$. The Mehler–Fock transform involving $P_{-1/2+iv}$ is a kind of hyperbolic version of the Fourier transform, and then the following result has the same flavor as some old results by Bochner and other authors (e.g., [19, 20]) in the Euclidean setting.

### Corollary 9

Let *F* be a completely monotonic function such that $F(x)=O\big (x^{-\sigma }\big )$ for some $\sigma >5/4$. Then

$$\begin{aligned} \int _{0}^\infty F\left( \frac{1}{4}+v^2\right) P'_{-1/2+iv}(\cosh r) v\tanh (\pi v)\, dv \le 0 \quad \text {for}\ r>0. \end{aligned}$$

### Proof

The heat kernel in the hyperbolic plane ${\mathbb {H}}^2$ is (see [21, (3.37)])

$$\begin{aligned} \frac{1}{2\pi } \int _{0}^\infty e^{-(\frac{1}{4}+v^2)t} P_{-1/2+iv}(\cosh r) v\tanh (\pi v)\; dv. \end{aligned}$$

Again, Bernstein’s theorem allows to replace the exponential by $F\big (\frac{1}{4}+v^2\big )$ keeping the monotonicity of the result, assured by Theorem 3 or Theorem 1, which must have a nonpositive derivative. The convergence of the integral and the differentiation under the integral sign is justified thanks to [16, 8.723.1], with the trivial estimate *O*(1) for the hypergeometric function, and the fact that $\Gamma (iv)/\Gamma (1/2+iv)\sim v^{-1/2}$ leads to $P'_{-1/2+iv}=O(v^{1/2})$. $\square$

Let us now proceed to the observation regarding local representations for fractional operators. Recently, great attention has been paid on the study of problems involving fractional and non-local operators. In particular, the fractional Laplace–Beltrami operator on manifolds appears in the study of different type of models such as free-boundary problems [22], porous medium equations [23], the Yamabe problem [24] and game theory, as they are related to stochastic Lévy flights [25]. In the Euclidean space or tori, it is a well-known consequence of Fourier analysis that the fractional Laplacian can be written as a singular integral operator (cf. [26]) which combined with elementary algebra provides useful pointwise estimates (cf. [27]). In the case of compact manifolds, an explicit integral representation for the fractional Laplace–Beltrami operator was only recently shown in [28] (cf. [12, 29] for the sphere). This new representation turned out to be essential in the proof of global existence of solutions to the surface quasi-geostrophic equation on the sphere (cf. [30]). Unfortunately, compared with the Euclidean case, the explicit representation contains an error term that makes it less powerful when pointwise control is needed, cf. [28]. As a consequence, several properties that are *straightforward* to check using the integral representation in the Euclidean case, as the maximum principle property or the positivity of the kernel, require more involved proofs. However, in the case of the unit sphere ${\mathbb {S}}^n$, an application of the decreasing property of Theorem 1 provides the following remark

### Corollary 10

Let $\alpha \in (0,1)$. There is a function $k_{n,\alpha }\,:\,[0,\pi ]\longrightarrow {{\mathbb {R}}}^+$ such that $k_{n,\alpha }(0)=1$, $d(x,y)^{-n-\alpha }k_{n,\alpha }(d(x,y))$ is decreasing for $d(x,y)\in (0,\pi )$, and

5 $$\begin{aligned} (-\Delta _{{\mathbb {S}}^n})^{\alpha }f(x)=c_{n,\alpha }\text {P.V.} \int _{{\mathbb {S}}^n}\frac{f(x)-f(y)}{d(x,y)^{n+2\alpha }}k_{n,\alpha }(d(x,y)) \; d\!{\text {vol}}(y). \end{aligned}$$

The main improvement of this result in comparison with the one obtained in [28] for general compact manifolds, where the operator is expressed as a similar principal value up to an operator that gains derivatives, is the absence of the latter.

### Proof

By the definition the heat kernel *G*(*x*, *y*, *t*), we have that

6 $$\begin{aligned} e^{-t{\Delta _{{\mathbb {S}}^n}}}f(x)=\int _{{\mathbb {S}}^n} G(x,y,t)f(y)\; d\!{\text {vol}}(y), \text{ and } \int _{{\mathbb {S}}^n} G(x,y,t)\; d\!{\text {vol}}(y)=1. \end{aligned}$$

Using the semigroup-formula (cf. [12, Section 7.1])

7 $$\begin{aligned} (-\Delta _{{\mathbb {S}}^n})^{\alpha }f(x)=\dfrac{1}{\Gamma (-\alpha )}\int _0^{\infty }\left( f(x)-e^{-t\Delta _{{\mathbb {S}}^n}}f(x)\right) \frac{dt}{t^{1+\alpha }}, \end{aligned}$$

combined with (6) and changing the order of integration it yields

8 $$\begin{aligned} \lim _{\epsilon \rightarrow 0}\int _{{\mathbb {S}}^{n}\setminus B_{\epsilon }(x)}(f(x)-f(y))\int _{0}^{\infty } G(x,y,t) \dfrac{dt}{t^{1+\alpha }} \; d\text {vol}(y). \end{aligned}$$

Notice that in order to apply Fubini’s theorem, we have subtract a small ball of radius $\epsilon$ with $\epsilon \rightarrow 0^+$. This limit is the principal value from the statement. For large times, the heat kernel is comparable to a constant, this is $c \le G(x,y,t) \le C$ (cf. [4]). Alternatively, one can show the upper bound using Hörmander’s $L^{\infty }$ bound coupled with the exponential decay of the coefficients. To deal with small times, one can either invoke the sharper estimate for *G*(*x*, *y*, *t*) in [4, Section 1] or use the heat kernel parametrix expansion (cf. [30, Section 2], [2, Section 6] or [31, Section 1]) to check that the singularity in (8) is of order $-n-2\alpha$. Combining both ingredients one can readily obtain

$$\begin{aligned} \int _{0}^\infty G(x,y,t) \dfrac{dt}{t^{1+\alpha }}= \dfrac{k_{n,\alpha }(d(x,y))}{d(x,y)^{n+2\alpha }} \end{aligned}$$

for some function $k_{n,\alpha }$ which only depends on the distance *d*(*x*, *y*), *n* and $\alpha$. Moreover, we can assure that *G*(*x*, *y*, *t*) is positive and decreasing as a function of $d(x,y)\in (0,\pi )$ by Theorem 1 applied to the sphere. $\square$

### Remark 7

Following the ideas sketched in Corollary 10, an interesting question is whether this observation could be extended to cover the case of compact hypersurface of revolution (as an application of Theorem 2) or complete spherically symmetric manifold with bounded curvature (as an application of Theorem 3).

## Final remarks

If the manifold is not spherically symmetric, one cannot hope a monotone behavior on the geodesic distance. For instance, for the flat torus ${{\mathbb {R}}}^2/({{\mathbb {Z}}}\times L{{\mathbb {Z}}})$ with $L>1$, the heat kernel based on the origin is

$$\begin{aligned} \frac{1}{L} \sum _{n,m\in {{\mathbb {Z}}}} e^{-4\pi ^2 t(n^2+m^2/L^2)+2\pi i(nx+my/L)} = \frac{1}{4\pi t} \sum _{n,m\in {{\mathbb {Z}}}} e^{-\big ((n-x)^2+(Lm-y)^2\big )/4t}, \end{aligned}$$

where the equality follows from the Poisson summation formula. It is apparent that it decays faster in the second coordinate. On the other hand, in this example we can separate the variables and for *y* fixed the kernel decreases when $0<x<1/2$ and, in the same way, it decreases when $0<y<L/2$ for *x* fixed. Any radial derivative is a combination of these, which shows that the heat kernel on a torus is decreasing in any radial direction departing from the origin up to the boundary of the fundamental domain.

It seems natural to try to decide whether there is a region with this monotonicity property in an arbitrary manifold. Notice that, in such a case, the region would depend on the initial point due to the possible lack of symmetries. An inspection of the sphere and the standard torus seems to suggest that, for every point, the monotonicity should hold up to its cut locus. This might be a by-product of some wishful thinking. On the other hand the sphere is, in some sense, the worst case scenario because the south pole is heated through every geodesic coming from the north pole. Perhaps an interesting context to start the study of these difficult questions is that of the hyperbolic Riemann surfaces, defined by $\Gamma \backslash {\mathbb {H}}^2$ with $\Gamma$ a Fuchsian group. The constant curvature and the connection with the arithmetic theory of automorphic forms could be of some help in some compact and noncompact examples. As a matter of fact, the heat kernels for tori are theta functions, which are prominent examples of automorphic forms.

## Footnotes

1 This formula also works in a broader generality for 3-dimensional space forms ${\mathbb {M}}_k$ with sectional curvature *k*.

2 This kind of manifolds are called “model manifolds with $R_0=\infty$” in [6] although we shall not employ this terminology here.

3 Recall that, for example, any function $u:(0,L)\times (0,+\infty )\rightarrow {{\mathbb {R}}}$ whose up to first order weak derivatives are locally square integrable and which solves (1) in the weak sense is indeed of class $C^\infty$ in $(0,L)\times (0,+\infty )$; see for instance [8, Theorem 7 in page 390] and the comments below it. Hence, the smoothness assumption is not really necessary here, we introduce it only for the sake of simplicity in the exposition.
